# Recurrent Pyogenic Granuloma Progressing to Calcifying Fibroblastic Granuloma in an Adolescent Male Athlete: A Case Report

**DOI:** 10.7759/cureus.77794

**Published:** 2025-01-21

**Authors:** Eliane Porto Barboza, Beatriz Panariello, Daniel Araujo, Diogo Rodrigues, Alexandra Manibo

**Affiliations:** 1 School of Dental Medicine, Lake Erie College of Osteopathic Medicine, Bradenton, USA; 2 Department of Periodontics, National Institute of Dental Science (INCO25), Niteroi, BRA

**Keywords:** calcifying fibroblastic granuloma, dental biofilm, oral health in athletes, oral pathology, peripheral ossifying fibroma, plaque accumulation, professional baseball players, pyogenic granuloma, teenage athlete

## Abstract

This case report highlights the clinical progression of a recurrent pyogenic granuloma (PG) in a 17-year-old male baseball player who experienced the transition of the lesion into a calcifying fibroblastic granuloma (CFG) extending into the periosteum and underlying bone. Adolescence, a period marked by significant hormonal changes and increased susceptibility to stress due to the demands of being an athlete, combined with a lack of proper oral hygiene, may have played a significant role in the lesion’s development, recurrence, and progression to CFG. The initial treatment involved excisional biopsy, followed by more extensive surgical intervention, including excision of the periosteum and bone curettage, to ensure complete removal of the recurrent lesion. The surgical site healed without complications. This case underscores the importance of accurate diagnosis and the need for thorough excision to prevent recurrence. Furthermore, it highlights the significance of maintaining proper oral hygiene and managing risk factors such as stress, which can influence the recurrence and progression of PGs to CFGs.

## Introduction

Pyogenic granuloma (PG), also known as lobular capillary hemangioma, is a common tumor-like pathology of the oral cavity [1-3]. It typically forms on the mucosal surface, especially in response to local irritation or trauma, and does not generally involve deeper tissues such as the periosteum or underlying bone. It usually presents as a smooth or lobulated mass that is highly vascularized [1-3]. The vascularization gives the lesion its reddish-purple color and explains the likelihood of bleeding associated with this type of lesion, even though they are usually painless [1]. These lesions most commonly appear in the maxillary anterior area of children, young adults, and pregnant women [1-3]. The histological characteristics of PG appear as channels lined with endothelium filled with red blood cells, featuring an ulcerated surface infiltrated by neutrophils [1]. As the lesion matures, it can progress and become more fibrotic [1,4]. The ideal treatment involves excising the lesion and scaling and root planing the adjacent teeth. The recurrence rate can be as high as 15%, and untreated lesions may eventually develop into calcifying fibroblastic granuloma (CFG) [2].

CFG, also known as peripheral ossifying fibroma, ossifying fibroid epulis, or peripheral fibroma with calcification, is an overreaction to external stimuli such as trauma or local irritants in the oral cavity [1,2,4]. However, unlike PG, CFG is more likely to extend into deeper tissues, including the periosteum and underlying bone [2,4-6]. CFG can develop from PG through fibrous maturation and calcification [1,4]. The mineralized calcifications originate from osteoprogenitor cells of the periodontal ligament or the periosteum [1,4]. The best way to differentiate PG from CFG is through histopathological evaluation as the clinical findings are too similar [1,4,6]. Histological features of CFG show fibrous connective tissue with variable fibroblast, collagen content, and mineralized material [1,4,6]. Mineralized materials can be bone, dystrophic calcifications, or cementum [1,4,6], especially if the lesion has been present for a longer duration or in areas of chronic irritation, such as near dental calculus or mature dental plaque (e.g., biofilm) [1,2,4,6].

Combined with systemic factors such as hormonal fluctuations, chronic stress, and nutritional deficiencies, bad oral hygiene can impair immune function and alter the body's inflammatory response to dental biofilm [3,7,8]. Puberty involves significant hormonal changes, especially a rise in testosterone levels in males, which contributes to increased inflammation of the gingival tissues [9]. Our patient, a professional baseball player, may also experience a weakened immune system and elevated cortisol levels due to physical stress and high training demands [10]. The combination of hormonal changes, immune dysfunction, elevated cortisol, and inadequate oral hygiene nurtures an environment favorable to oral dysbiosis [7,8], worsening the risk of developing oral pathologies.

The surgical treatment of PG and CFG significantly differ when considering the periosteum involvement because of these lesions’ distinct pathophysiology and tissue characteristics [6]. In surgical therapy for PG, the main goal is to excise the lesion completely, but as it rarely invades the periosteum, the procedure focuses on removal from the soft tissue. On the other hand, surgical treatment of CFG requires deeper excision to ensure complete removal. It is crucial to remove both the lesion and any affected periosteal tissue or bone [6]. Due to both lesions most likely being caused by trauma or local irritants such as dental biofilm and calculus [1,2,6,11,12], the best course of action for the prevention of recurrence is to educate the patient about proper oral hygiene and maintain good compliance with their professional dental cleanings.

This clinical case report presents a recurrent PG that progressed to a CFG in a 17-year-old European male baseball player.

## Case presentation

**Figure 1:**
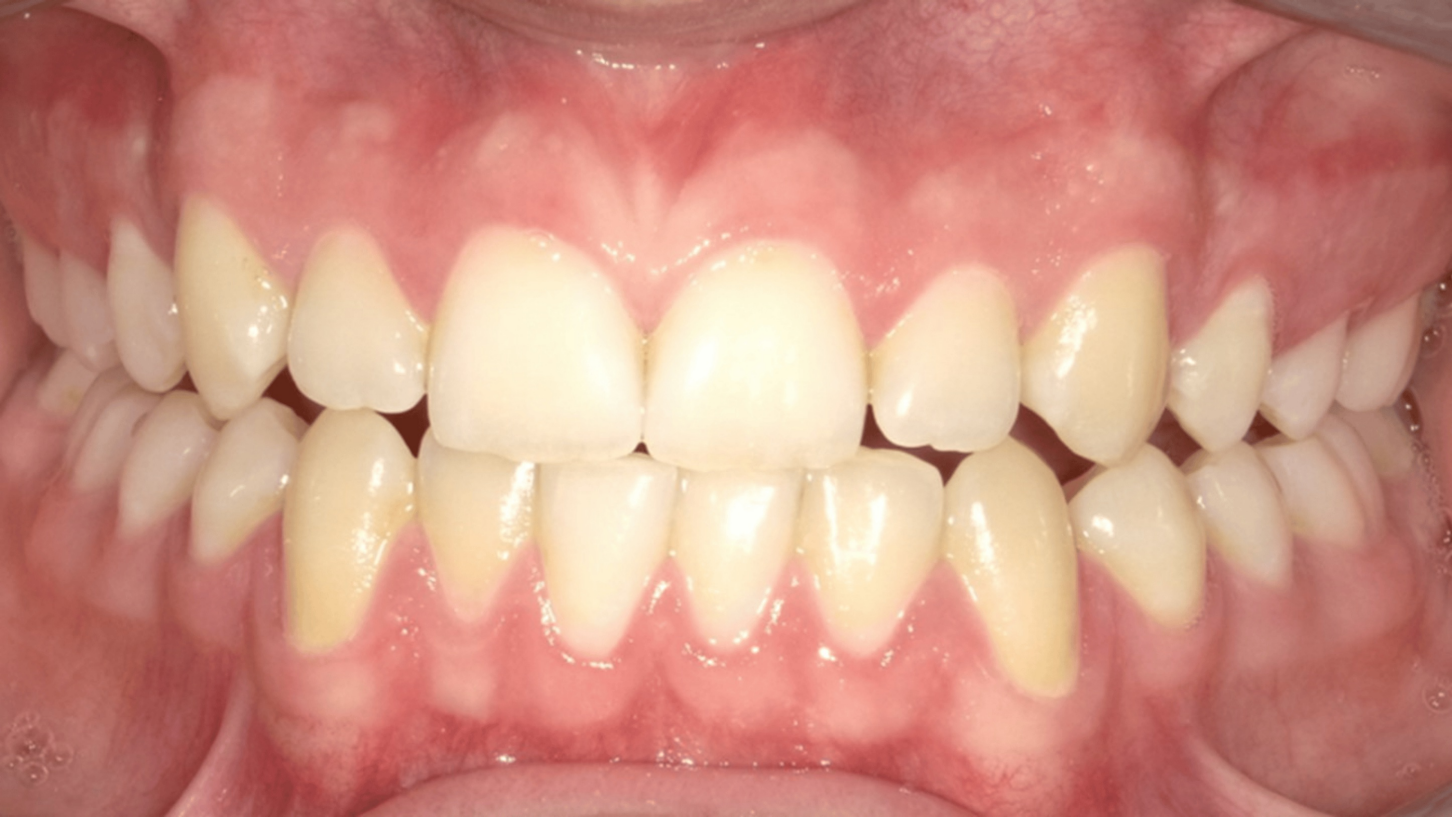
Upon the patient’s initial visit, the examination revealed a healthy periodontium with no signs of active disease or inflammation.

A 17-year-old male European baseball athlete presented to the Lake Erie College of Osteopathic Medicine, School of Dental Medicine clinic in Bradenton, FL, on April 11, 2018, for a routine dental cleaning. The patient reported no dental pain or discomfort at this time. After clinical evaluation, he was diagnosed as having a healthy periodontium, and no significant findings in the oral cavity were found. Adult prophylaxis and oral hygiene reinforcement were performed. Two years later, the patient returned for a complete oral evaluation. A panoramic radiograph and a full mouth X-ray were taken, which showed no abnormalities, and clinical findings confirmed a healthy periodontium (Figure 1).

**Figure 2:**
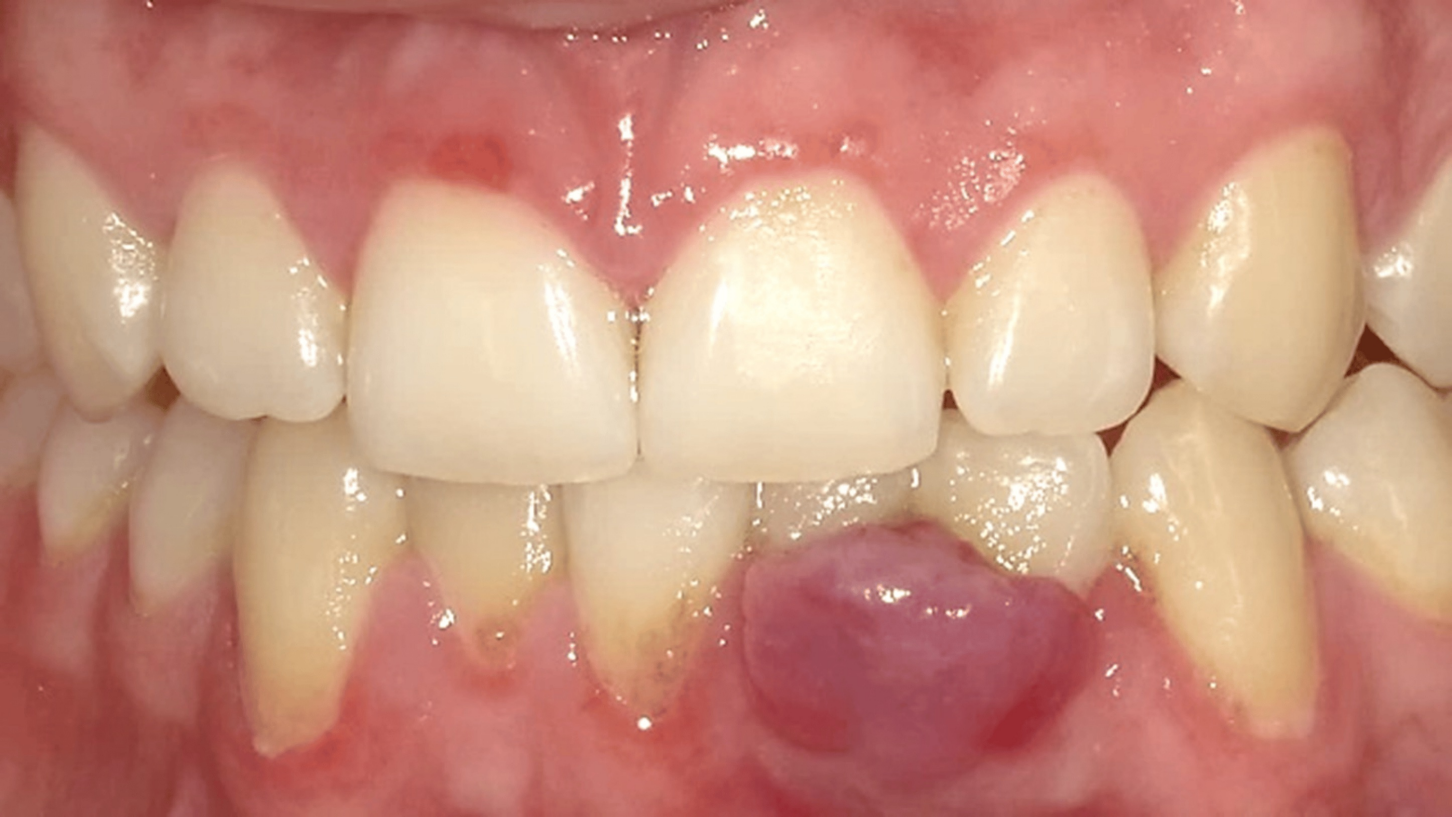
Patient presented to the clinic with concerns about a nodule on the gingiva. Upon examination, there was a notable increase in dental biofilm accumulation compared to the previous appointment. Clinical evaluation suggested a possible diagnosis of pyogenic granuloma, based on the appearance of the lesion, irritation caused by biofilm accumulation, and surrounding tissue changes.

Six months later, the patient presented to the clinic with complaints of a rapidly growing mass in his gingiva that was both uncomfortable and aesthetically displeasing. Clinical examination showed no signs of trauma in the affected area, and radiographic evaluation indicated no bone loss. The lesion displayed features suggestive of a possible PG, leading to a referral for an oral pathology consultation. Located in the mandibular arch, the lesion was described as a small, lobulated, red nodule found in the gingival space between the left inferior central incisor and the left inferior lateral incisor. The patient underwent an excisional biopsy, and the tissue samples were sent to the Oral and Maxillofacial Pathology Laboratory at the University of Florida College of Dentistry for histopathological evaluation. The results confirmed a diagnosis of PG. Postoperative care instructions were provided, emphasizing the importance of maintaining oral hygiene to prevent recurrence. One year later, the patient returned with a recurrence of the lesion and generalized plaque-induced gingivitis, raising concerns regarding the patient’s compliance with home care (Figure 2).

**Figure 3:**
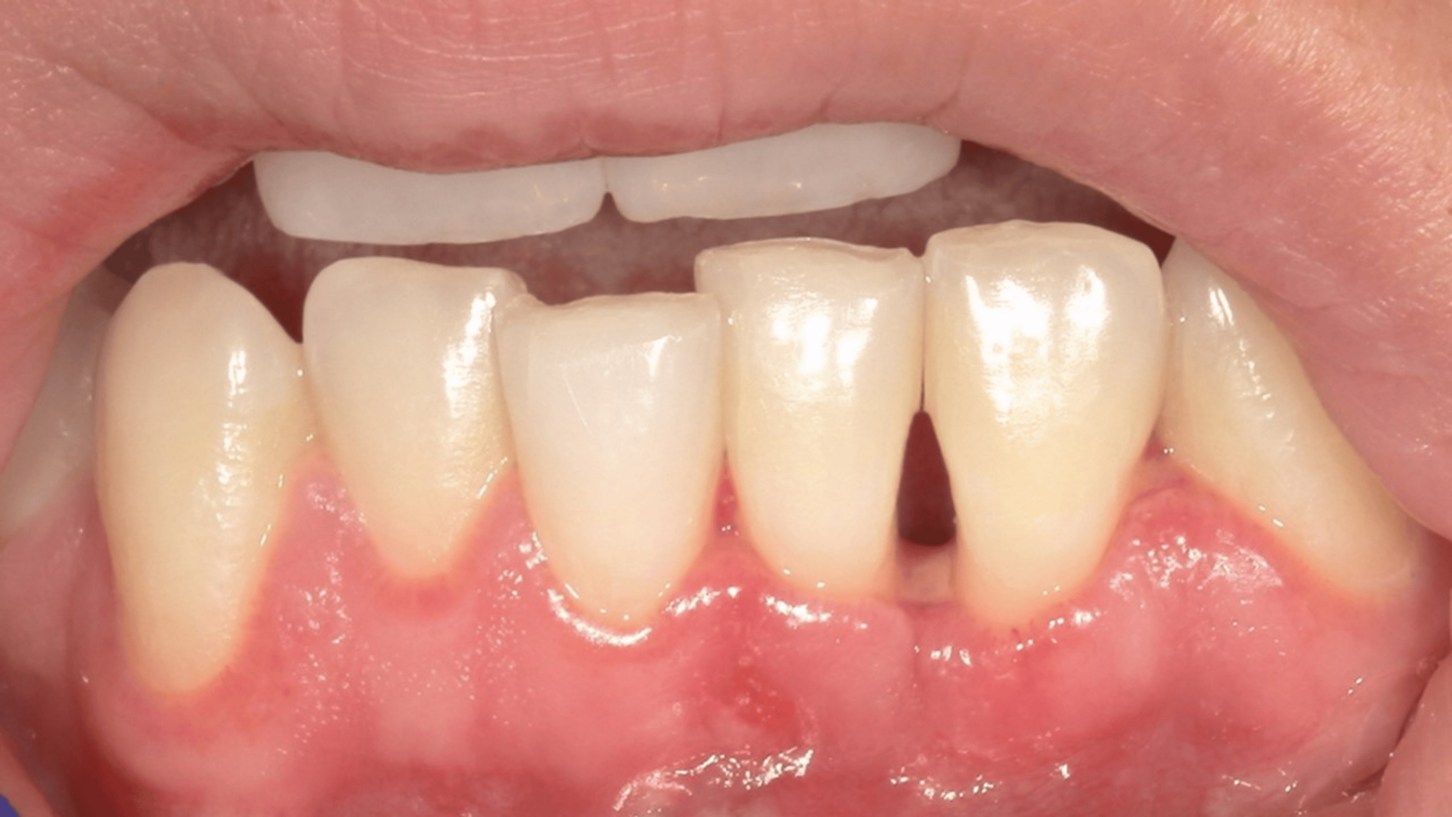
Postoperative image. Healing was uneventful. Note the black space between the left inferior central incisor and the left inferior lateral incisor, indicating clinical attachment loss.

A full-mouth adult prophylaxis and reinforced oral hygiene instructions were performed. A surgical intervention was then conducted to excise the recurrent lesion, and the healing process was uneventful (Figure 3).

**Figure 4:**
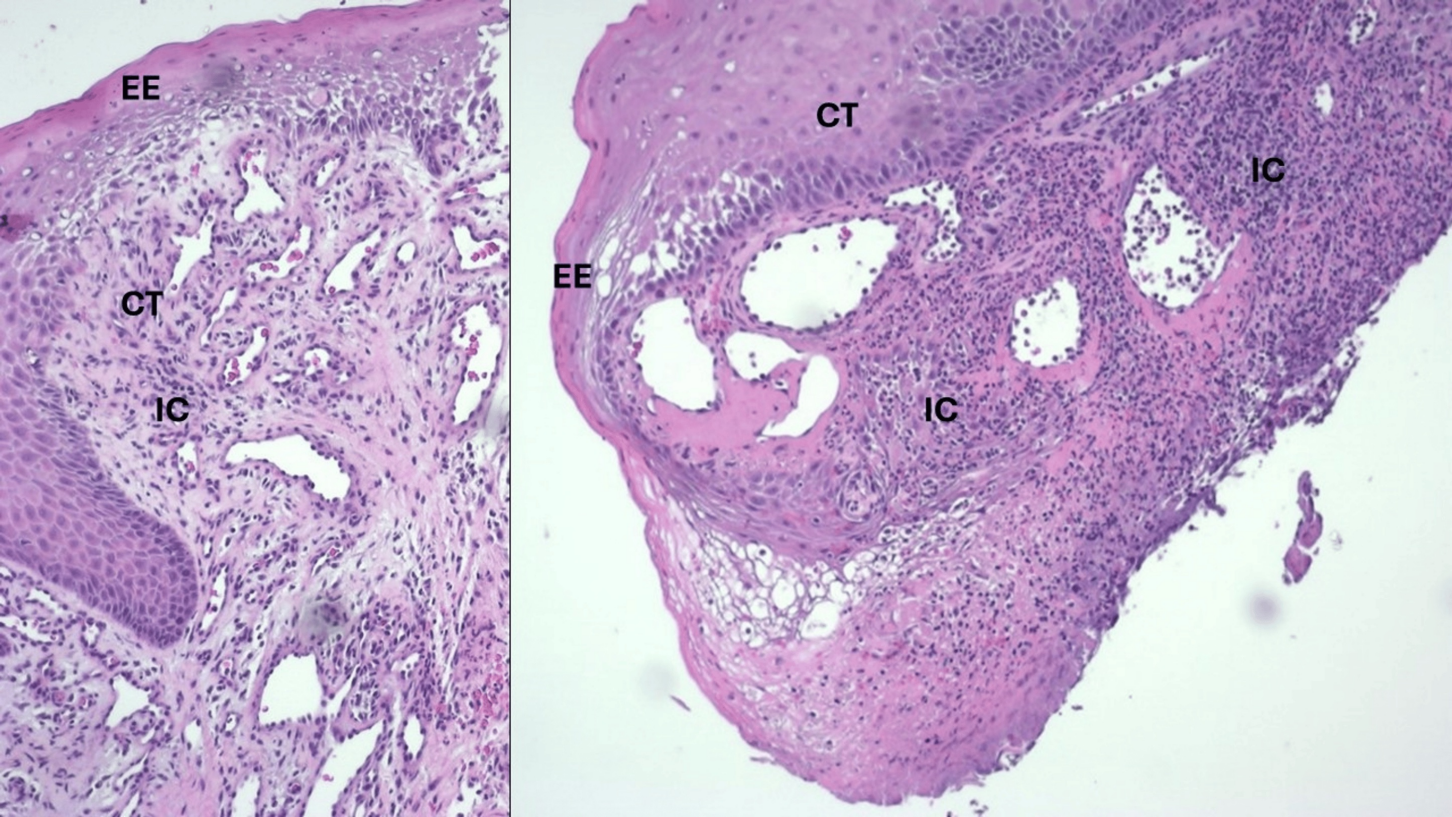
Histopathological analysis revealing keratinized stratified squamous epithelium (EE) overlying a mass of inflamed and vascular fibrous connective tissue (CT). The epithelium is covered by a thickened layer of frayed parakeratin. Large areas of epithelium are discontinuous where a fibrinous exudate intermixed with necrotic inflammatory cells is seen. The underlying fibrous connective tissue mass, which forms the bulk of the specimen, displays numerous dilated and engorged capillaries, fibroblasts, and endothelial cells. An intense inflammatory cell infiltrate (IC) consisting of lymphocytes, plasma cells, and neutrophils is seen through this framework. Diagnosis is consistent with pyogenic granuloma.

The samples were submitted to the Oral and Maxillofacial Pathology Laboratory at the University of Florida College of Dentistry for histopathological analysis, which confirmed a PG (Figure 4).

**Figure 5:**
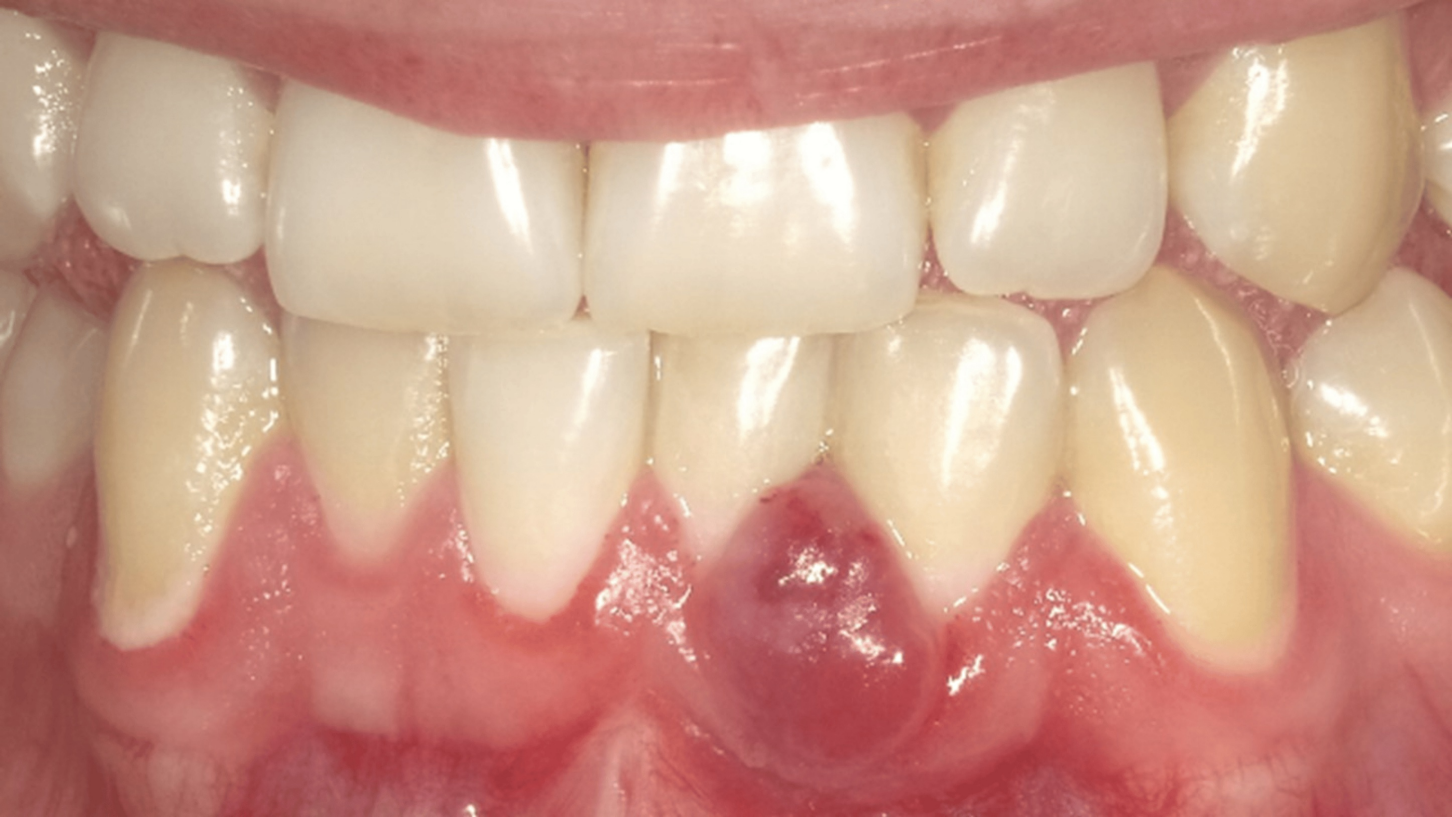
Recurrent lesion, firm on palpation.

Two months later, the patient was presented once more with a recurrent lesion (Figure 5).

**Figure 6:**
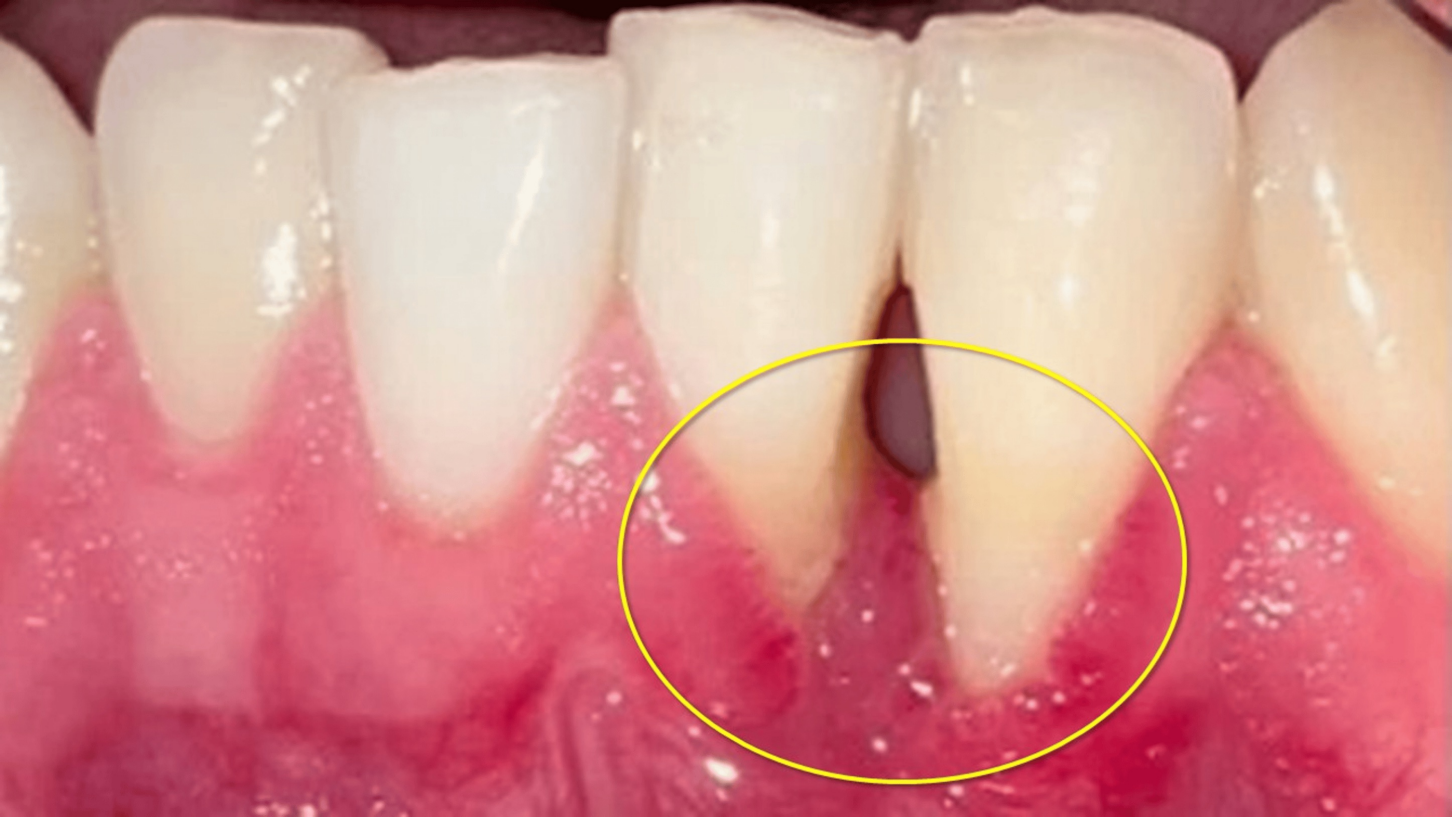
Two weeks postoperatively. Note the increase of attachment loss between the left inferior central incisor and the left inferior lateral incisor.

At this time, the lesion presented dense to palpation. Therefore, due to the recurrence of the lesion and its clinical presentation, we assumed the recurrent PG had progressed to CFG. An excisional biopsy was performed to remove the lesion and the surrounding periosteum. Bone curettage was also conducted. The sample was then sent to the Oral and Maxillofacial Pathology Laboratory at the University of Florida College of Dentistry for histopathological evaluation. During a follow-up appointment, healing at the surgical site was observed, with no complications reported (Figure 6).

**Figure 7:**
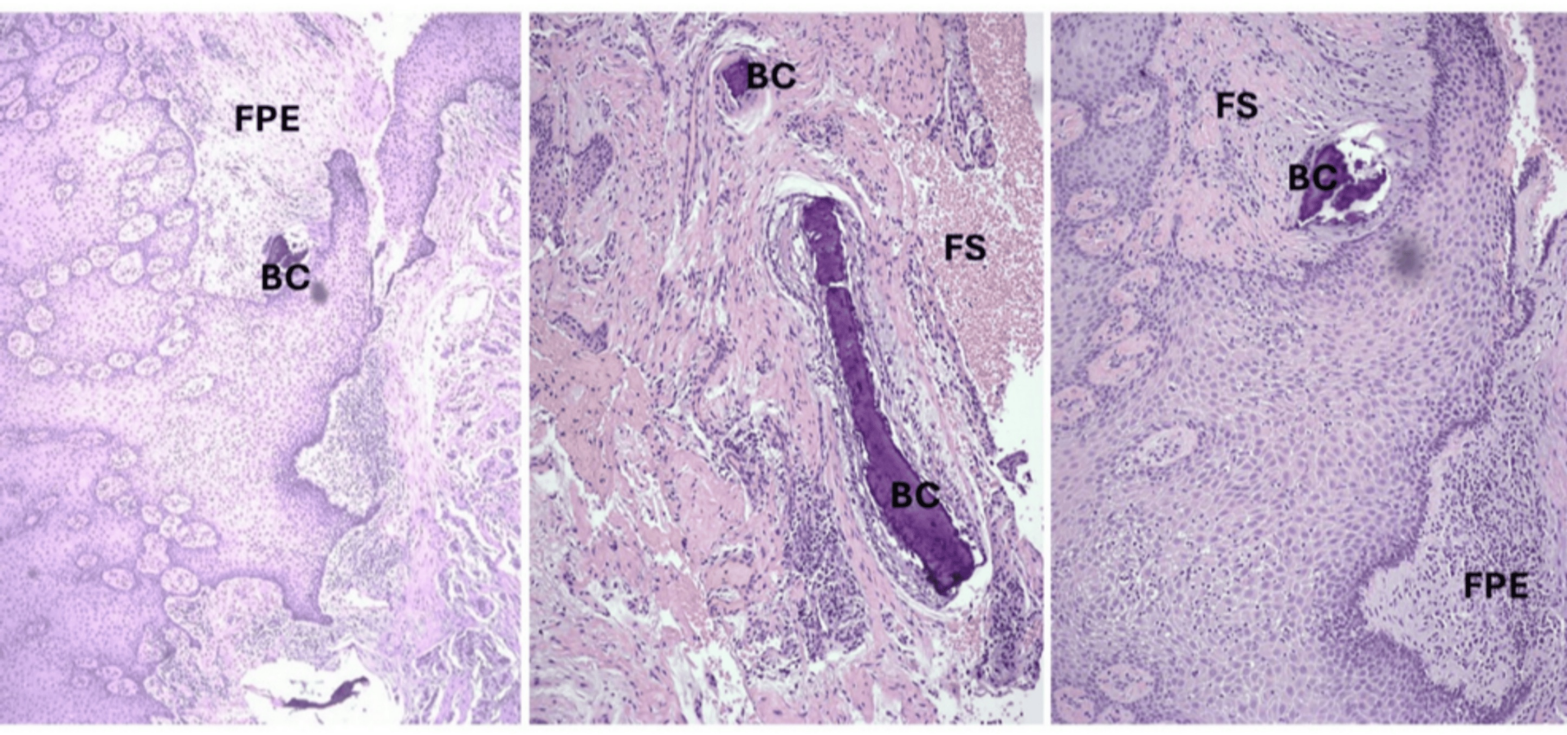
Histopathological analysis revealed a smooth surfaced mass composed of superficial stratified squamous epithelium and underlying fibrous connective tissue. Epithelium is within normal limits. The surface is ulcerated and eroded over a large area where it is covered by a fibropurulent exudate (FPE). The underlying fibrous connective tissue is composed of a cellular, fibroblastic stroma (FS) containing scattered variously sized and shaped small-sized basophilic calcifications (BC). Scattered chronic inflammatory cells are noted throughout the stroma. Diagnosis is consistent with peripheral ossifying granuloma, ulcerated.

After the treatment, the patient returned to Europe and was advised to maintain good oral hygiene to prevent future issues. Periodontal plastic surgery could be considered to gain keratinized tissue. The patient was encouraged to follow up with a dentist in his home country. Histopathological images obtained from the recurrent lesion biopsy confirm the CFG diagnosis (Figure 7).

## Discussion

This case report highlights the clinical progression of a 17-year-old male baseball player who developed a recurrent gingival lesion that transitioned from a PG to a CFG. Gingival lesions such as PGs are relatively common, and their occurrence is often associated with localized trauma, irritation, or poor oral hygiene [1,2,6,11,12]. A review of 215 cases over 20 years identified gingival irritation and inflammation due to poor oral hygiene as the primary contributing factors for developing PGs [12]. Similarly, a study that reviewed 94 PG cases found that over 87% of the cases were associated with inadequate oral hygiene [11]. Poor oral hygiene leads to mature dental biofilm and calculus accumulation, which fosters an inflammatory environment in the periodontal tissues [3,13]. The relationship between inadequate oral hygiene and the development of reactive gingival lesions is well-established [1-3], as the irritation from mature dental plaque (e.g., biofilm), calculus, and inflamed gingival tissues provides a continuous stimulus for abnormal tissue growth [1,3,11-13]. These issues, when combined with systemic factors, such as major hormonal changes, chronic stress, and nutritional imbalances, can compromise the immune system and alter the body’s inflammatory response to dental biofilms [7,8,14].

Adolescence is characterized by significant hormonal changes, with a peak in male circulating testosterone. Elevated levels of sex hormones during this time have been associated with increased gingival inflammation [9]. Additionally, our patient is an athlete, and it has been reported that athletes may develop a weakened immune system and elevated cortisol levels due to stress, physical demands, and overload [10]. Hormonal changes, impaired immune function, elevated cortisol levels, and poor oral hygiene contribute to dysbiosis in the oral environment [3,7,14] and place the patient at a higher risk for developing oral pathologies [8-10,14]. We believe that the combination of these factors may have contributed to the recurrent episodes of PG and, eventually, to the progression of PG to CFG.

It is known that PGs tend to recur, particularly if not excised entirely or if the underlying cause of irritation persists [1,5,6]. Possibly the initial excision did not thoroughly remove all affected tissue. In addition, persistent biofilm accumulation provided a continuous irritative stimulus to the periodontal tissues [1,3,13], which, along with the systemic factors mentioned above (e.g., hormonal changes and stress), caused a dysbiotic oral environment [7,8]. Therefore, the combination of these factors may have contributed to the transformation of the lesion into a more fibrous and calcified form, known as calcifying fibroblastic granuloma. Unlike PGs, CFGs can involve the underlying bone [1,2,4-6]. CFGs often arise from the periodontal ligament or gingival connective tissue, typically in response to chronic irritation from biofilm accumulation or trauma [2,4,6]. They were found to be more commonly observed in female patients during their third and fourth decades of life [6]; however, the present case report contrasts with this pattern, as it involves a 17-year-old male patient.

The treatments applied throughout this case were based on sound clinical principles for managing reactive gingival lesions. The initial excisional biopsy of the PG was appropriate for obtaining a diagnosis and alleviating the discomfort caused by the lesion. This approach also allowed for histopathological evaluation, confirming the diagnosis of PG.

Upon the third recurrence of the lesion, clinical examination revealed a denser, more fibrous nodule, suggesting possible calcification. A more extensive surgical removal of the lesion was then performed, which included the excision of both the lesion and the surrounding periosteum, along with bone curettage. This was done to ensure the complete removal of the reactive tissue and any calcified material that may have contributed to the progression of the lesion. In addition to surgical treatment, reinforcing the importance of proper oral hygiene was critical to the treatment plan. As inadequate oral hygiene is a significant factor contributing to the recurrences, providing the patient with oral hygiene instructions and emphasizing the need for regular professional cleaning and plaque control at home is essential in preventing further recurrences. In the future, periodontal plastic surgery may be considered to enhance keratinized tissue and improve aesthetics.

In this clinical case, we observed that factors such as poor oral hygiene, hormonal changes, and chronic stress contributed to the recurrence of PGs and the progression to CFG. The treatments applied, including excisional biopsy, periosteal removal, and bone curettage, were appropriate for managing the lesions and aimed at reducing the risk of recidivism. However, patient compliance with maintaining good oral hygiene at home and attending regular follow-up appointments with the dentist is essential to prevent the reappearance of the lesions.

## Conclusions

This case report underscores the clinical significance of recognizing the potential progression of PG to CFG, particularly in adolescents. The case highlights the complexities associated with recurrent PGs, which may evolve into more invasive lesions involving the periosteum and underlying bone, requiring extensive surgical intervention. Clinicians must be vigilant in their diagnosis and ensure complete excision to prevent recurrence and minimize the risk of further complications. Additionally, understanding the contributing factors, such as hormonal changes, physical stress, and inadequate oral hygiene, is crucial in managing PGs. Education on proper oral hygiene, stress management, and preventive measures should be emphasized to reduce the recurrence and progression of PGs to CFGs. This case advocates for a multidisciplinary approach to diagnosis and treatment, focusing on early detection, thorough surgical management, and patient education to optimize outcomes and prevent complications.
